# Beneficial effects of Oltipraz, nuclear factor - erythroid – 2 - related factor 2 (Nrf2), on renal damage in unilateral ureteral obstruction rat model

**DOI:** 10.1590/S1677-5538.IBJU.2018.0232

**Published:** 2018

**Authors:** Emre Can Polat, Huseyin Besiroglu, Levent Ozcan, Alper Otunctemur, Ahmet Tugrul Eruyar, Adnan Somay, Nurver Ozbay, Mustafa Cekmen, Ceyla Eraldemir, Emin Ozbek

**Affiliations:** 1 Department of Urology, Okmeydani Training and Research Hospital, University of Health Sciences, Istanbul, Turkey;; 2 Department of Urology, Catalca Ilyas Cokay State Hospital, Istanbul, Turkey;; 3 Department of Urology, Derince Training and Research Hospital, University of Health Sciences, Kocaeli, Turkey;; 4 Department of Pathology, Derince Training and Research Hospital, University of Health Sciences, Kocaeli, Turkey;; 5 Department of Pathology, Fatih Sultan Mehmet Training and Research Hospital, University of Health Sciences, Istanbul, Turkey;; 6 Department of Biochemistry, Istanbul Medeniyet University, Istanbul, Turkey;; 7 Department of Biochemistry, Kocaeli University, Kocaeli, Turkey;; 8 Department of Urology, Cerrahpasa Medical School, Istanbul University, Istanbul, Turkey

**Keywords:** Oltipraz [Supplementary Concept], Renal Insufficiency, Ureter

## Abstract

**Introduction:**

We investigated whether Oltipraz (OPZ) attenuated renal fibrosis in a unilateral ureteral obstruction (UUO) rat model.

**Materials and Methods:**

We randomly divided 32 rats into four groups, each consisting of eight animals as follows: Rats in group 1 underwent a sham operation and received no treatment. Rats in group 2 underwent a sham operation and received OPZ. Rats in group 3 underwent unilateral ureteral ligation and received no treatment. Group 4 rats were subjected to unilateral ureteral ligation plus OPZ administration. Transforming growth factor beta-1 (TGF-β1), E-cadherin, nitric oxide (NO) and hydroxyproline levels were measured. Histopathological and immunohistochemical examinations were carried out.

**Results:**

TGF-β1, NO and E-cadherin levels in the UUO group were significantly higher than the sham group and these values were significantly different in treated groups compared to the UUO group. In rats treated with UUO + OPZ, despite the presence of mild tubular degeneration and less severe tubular necrosis, glomeruli maintained a better morphology when compared to the UUO group. Expressions of α-SMA in immunohistochemistry showed that the staining positivity decreased in the tubules of the OPZ-treated group.

**Conclusions:**

While the precise mechanism of action remains unknown, our results demonstrated that OPZ exerted a protective role in the UUO-mediated renal fibrosis rat model highlighting a promising therapeutic potency of Nrf2-activators for alleviating the detrimental effects of unilateral obstruction in kidneys.

## INTRODUCTION

Ureteral obstruction occurs at any stage of life from fetal development to adulthood in any segment of the ureter between ureteral orifices and the renal pelvis. The histopathologic changes are characterized by tubular dilatation or atrophy, inflammatory cell infiltration, fibroblast activation and proliferation, increases in matrix proteins, and progressive tubulointerstitial fibrosis. These histopathologic processes might eventually result in the loss of renal parenchyma leading to permanent renal function deterioration.

One of the most prevalent molecular mechanisms of tubulointerstitial fibrosis due to ureteral obstruction is epithelial - to - mesenchymal transition. Numerous studies have proposed that under pathologic conditions renal tubular epithelial cells may undergo a phenotypic transformation into matrix - producing myofibroblasts by an epithelial - mesenchymal transition (EMT) process (1-3). Activated by several growth factors such as transforming growth factor β (TGFβ), myofibroblasts function as the primary source for producing extracellular matrix (ECM), including collagen and fibronectin. Furthermore, the expression of intercellular epithelial adhesion molecules such as E - cadherin decrease and mesenchymal cell markers such as α - smooth muscle actin (α - SMA), N - cadherin and vimentin increase. For this reason, EMT is recognized as a molecular component of renal fibrosis (4-6).

Reactive oxygen species (ROS) have a major role in the development of renal fibrosis inducing epithelial - mesenchymal transition (EMT) in the presence of UUO. Nitric oxide (NO), a vasodilator, has been implicated in late renal hemodynamic changes and observed upregulated in the kidney during the UUO process. Even though the excessive amounts of nitric oxide (NO) are oxidized into ROS, they have proven to be useful in interrupting signaling and controlling inflammation (7, 8). In addition, renal hydroxyproline (Hyp), which is commonly used to measure the collagen content in biological tissue values, has been used to assess fibrosis (9).

Oltipraz (OPZ), 5 - (2 - pyrazynyl) – 4 - methyl – 1, 2 - dithiole – 3 - thione, is a synthetic dithiolethione that targets Nrf2 (nuclear factor - erythroid – 2 - related factor 2), an agent that plays a pivotal role in cellular defense against oxidative stress by promoting the transcription of various antioxidant genes (10). Several agents have been used to prevent UUO - induced renal injury in animal models but there is no experiment for the role of oltipraz (OPZ) in the literature. A randomized, double-blind, placebo - controlled phase II trial demonstrated that reductions in inflammation, oxidative stress and fibrosis could be achieved using oltipraz (OPZ), nuclear factor - erythroid – 2 - related factor 2 (Nrf2) activator in patients with liver fibrosis or cirrhosis (11).

Based on these findings, we investigated the potential effect of OPZ in attenuating renal fibrosis induced by UUO in rats.

## MATERIALS AND METHODS

### Animals

Male Wistar albino rats, weighing 200 to 250 g and six to seven weeks old, were housed in clean plastic cages in a temperature - and humidity - controlled facility under constant 12 - hour light / 12 - hour dark photoperiods with free access to food and water. The Institutional Animal Care and Use Committee approved the use of animals and the experimental protocol and animals were treated according to the Guide for the Care and Use of Laboratory Animals of Research Council.

### Treatment and experimental protocols

One week after acclimatization, UUO was induced. Briefly, after induction of general anesthesia by intraperitoneal injection of thiopental (100 mg / kg), the abdominal cavity was exposed via a midline incision and the left ureter was ligated at two points with 3-0 silk. The sham - operated rats had their ureters manipulated but not ligated. All rats were given amikacin sulfate (6 mg / kg, intramuscular route) before the operation (12). After a quarantine period of seven days, 32 rats were randomly divided into four groups, each consisting of eight animals as follows: Rats in group 1 underwent a sham operation and received no treatment. Rats in group 2 underwent a sham operation and received OPZ (Sigma Chemical Co., St. Louis, MO) (p.o. 30 mg / kg body weight / day). Rats in group 3 underwent unilateral ureteral ligation and received no treatment. Group 4 rats were subjected to unilateral ureteral ligation and received OPZ. The OPZ dose was based on the previous studies (13).

At 14 days after UUO, all rats were sacrificed by high - dose ketamine. Kidneys were reached with an abdominal midline incision. The left kidney was immediately excised and separated from the surrounding tissues, washed twice with cold saline, and stored at – 800 C to determine the markers of renal fibrosis, EMT and tubular injury.

A portion of the left renal tissue was stored in formol solution for the histopathologic and immunohistochemical examinations. Paraffinized tissue samples were examined for leukocyte infiltration and renal fibrosis.

Measurement of transforming growth factor beta – 1, E - cadherin, nitric oxide and hydroxyproline levels.

The TGFβ – 1 ELISA kit (ref MB100B, R & D Systems) was used to measure TGFβ – 1 in renal tissue 50 microns of total proteins from each tissue were assayed. This assay detects activated TGFβ-1 with a sensitivity of 4.6 pg / mL. The coefficient of variation intra-assay is < 4%, and the coefficient inter - assay is < 8%.

The concentration of soluble E - cadherin was measured with a commercially available sandwich enzyme - linked immunosorbent assay kit based on monoclonal antibodies (Zymed Laboratories Inc., CA). The coefficient of variation intra - assay is < 5%, and the coefficient inter - assay is 7%. The sensitivity is 2.0 ng / mL.

The nitrate concentrations in samples were assayed by enzymatically reducing nitrate. 50 microL of samples were incubated with the same volume of reductase buffer (0.1M) potassium adenine dinucleotide and four units of nitrate curve were obtained by incubating sodium nitrate (10 – 200 μM) with the buffer. The total amount of nitrite and the amount of nitrite in the samples was then determined using the Griess method (14). The samples were incubated with the same volume of Griess reagent (1% sulphanilamide and 0.1% naphthyl ethylenediamine dihydrochloride in 5% phosphoric acid). The absorbance at 550 nm was determined using a multiwell plate reader. The results were reported as the concentration of nitrate plus nitrite (microM NO3 + NO2) for samples of nitrite for supernatants.

Renal tissue fragments were homogenized in saline 0.9%, frozen and lyophilized. The assay was performed with 40 mg of the lyophilized tissue that was subjected to alkaline hydrolysis in 300 micros plus 75 microLNaOH 10 moL / L at 120 degrees C for 20 minutes. An aliquot of 50 microL of the hydrolyzed tissue was added to 450 microL of chloramine T oxidizing reagent (Chloramine T 0.056 moL / L, n - propanol 10% in acetate / citrate buffer pH 6.5) and allowed to react for 20 minutes. A hydroxyproline standard curve with the highest concentration of 400 microns was prepared in a similar fashion. The color was developed by the addition of 500 microL of the Ehrlich reagent (p - dimethylamine - benzaldehyde, 1 moL / L) diluted in n - propanol / perchloric acid, 2: 1 supernatant was transferred to 96 - well plates, and the absorbance was read at 550 nm.

### Histopathological and immunohistochemical examination

Histopathological evaluation was carried out on kidney tissues. Paraffin - embedded specimens cut into 6 - μm thickness sections were processed with hematoxylin and eosin stain for examination under the light microscope using a conventional protocol (15) (BH – 2; Olympus, Tokyo, Japan). Semi - quantitative evaluation of renal tissues was performed by scoring the degree of severity according to previously published criteria (16). All sections of kidney samples were examined for tubular necrosis. Briefly, a minimum of 50 proximal tubules associated with 50 glomeruli were examined for each slide and an average score was obtained. The severity of lesion was graded from 0 to 3 according to the percentage of tubular involvement. Slides were examined and assigned for severity of changes using scores on a scale in which (0) denotes no change, grade (1) changes affecting < 25% tubular damage (mild), grade (2) changes affecting 25 – 50% of tubules (moderate) and grade (3) changes affecting > 50% of tubules (severe).

The histopathological and immunohistochemical evaluation were performed on left kidney tissues. Paraffin - embedded specimens were cut into 5 - mm thick sections and stained with hematoxylin and eosin, Masson's trichrome and α - smooth muscle actin (α - SMA) were used for examination under the light microscope (BH – 2; Olympus, Tokyo, Japan).

To evaluate leukocyte infiltration, the widening of interstitial spaces with focal leukocyte infiltration was assessed in five randomly chosen sections prepared from each kidney sample. For each section, the average number of leukocytes per 0.28 mm^2^ was calculated from these leukocyte - infiltrated foci using a high - power microscopic field (x 400).

To estimate the grade of interstitial fibrosis, the interstitial area that was stained green with Masson's trichrome was evaluated as a percentage of the total examined area in five randomly chosen sections prepared from each kidney sample using an image analyzer (Leica; Leica Micros Imaging Solutions, Cambridge, UK). For each section, interstitial space widening with focal leukocyte infiltration and interstitial fibrosis was assessed in high - power fields (x 400) to quantify the results. The Banff classification of kidney pathology was used to score the degree of mononuclear cell infiltration and interstitial fibrosis. The score was graded from 0 to 3, depending on the severity of histological characteristics (17).

### Statistical analyses

Continuous variables of all groups were demonstrated as mean values ± standard deviation (SD). Statistical analyses of the histopathologic evaluation of the groups were carried out by the Chi - square test, and biochemical data among four groups were analyzed by Kruskal - Wallis test. The p - value of < 0.05 was accepted as statistically significant. In case a statistical significant was observed between three or four groups in Kruskal - Wallis test, Mann - Whitney U test was utilized for the detection of the significance between two groups.

## RESULTS

Transforming growth factor beta – 1, E - cadherin, nitric oxide and hydroxyproline levels TGF - β1 level in sham, UUO and UUO + OPZ groups were 2.5 ± 0.6; 7.2 ± 1.5 and 3.6 ± 0.9 respectively. NO level in sham, UUO and UUO + OPZ groups were 20.6 ± 3.5; 62.3 ± 19.8 and 37.4 ± 15.1 respectively and E - cadherin level in those groups were 3.7 ± 0.5; 7.4 ± 1.2 and 3.9 ± 1.8 respectively. TGF - β1, NO, and E - cadherin levels were significantly higher in the UUO group than the sham group. UUO + OPZ group had lower levels of these markers compared to the UUO group. Hydroxyproline level was 423.5 ± 63.8 in UUO group, whereas the remaining three groups had lower levels; but this difference did not reach a statistical significance. The details are shown in Table-1.

**Table 1:** TGF-β1, E-cadherin, NO and Hydroxyproline levels in kidney.

Parameters	Sham	Sham + OPZ	UUO	UUO+OPZ
TGF-β1(pg/ mL)	2.5 ± 0.6	2.7 ± 0.5	7.2 ± 1.5	3.6 ± 0.9*
E-cadherin(ng/ mL)	3.7 ± 0.5	3.5 ± 0.6	7.4 ± 1.2	3.9 ± 1.8*
NO(μmoL/g)	20.6 ± 3.5	19.5 ± 2.9	62.3 ± 19.8	37.4 ± 15.1*
Hydroxyproline (pg/mL)	357.5 ± 84.9	368.1 ± 51.8	423.5 ± 63.8	386.8 ± 62.1

**TGF =** transforming growth factor β1; **NO =** nitric oxide.

Values are expressed as mean ± SD for eight rats in each group.

* Significantly different from UUO group (p < 0.05).

**TGF-β1 =** NO and E-cadherin levels in UUO group were significantly higher than Sham group, in treated groups these values were significantly different from UUO group. Hydroxyproline levels were higher in UUO group, but results were not significant among other groups.

### Histopathologic and immunohistochemical examination results

**Figure 1:**
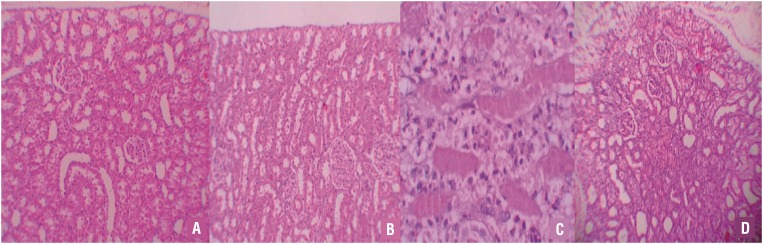
A) Normal tubulus and glomeruli in kidney cortex H&Ex100 (sham group). B) Normal tubulus and glomeruli in kidney cortex H&Ex100(sham+OPZ group). C) Severe tubular total necrosis, tubular degeneration and epithelial vacuolization in the proximal tubules H&Ex400(UUO group). D) Mild epithelial vacuolization in the proximal tubules and normal glomeruli H&Ex100 (UUO+OPZ treated group).

Histopathologic examination of kidneys showed no pathologic findings in the sham and sham + OPZ groups (Figures 1A and 1B). Mild and severe tubular necrosis in the proximal tubules was found in rats with UUO compared to the sham group (Figure-1C). Despite the presence of mild tubular degeneration and less severe tubular necrosis in rats treated with UUO + OPZ, glomeruli maintained a better morphology compared to the UUO group (Figure-1D).

**Figure 2:**
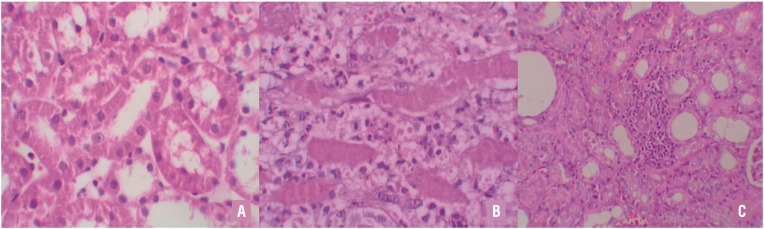
A) Normal kidney morphology in a sham group. B) Leukocyte infiltration was observed in the peritubular interstitium of the UUO. C) Leukocyte infiltration was reduced in the OPZ-treated group (hematoxylin&eosin,*400).

Histopathologic examination was normal in rats with the sham operation (group l). Severe leukocyte infiltration was observed in the periglomerular and peritubular interstitium of the rat kidneys in group 3 with UUO (Figures 2A and B). Quantitative analysis of the focal leukocyte infiltration area in the interstitium showed that leukocyte infiltration was significantly reduced in rats administered with UUO + OPZ (group 4) (Figure-2C).

**Figure 3:**
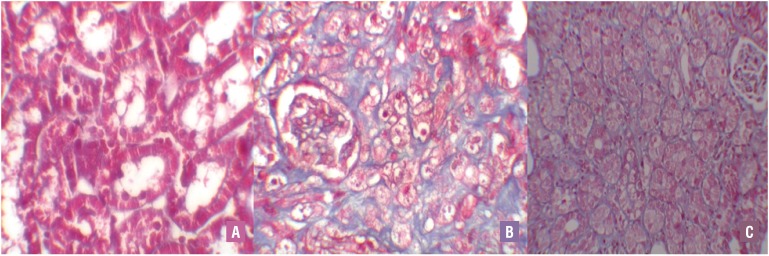
A) Normal kidney morphology in a sham group. B) Severe fibrosis was observed in the peritubular interstitium of the UUO. C) Mild fibrosis was reduced in the OPZ-treated group (Masson&Trichrome, *400).

**Figure 4:**
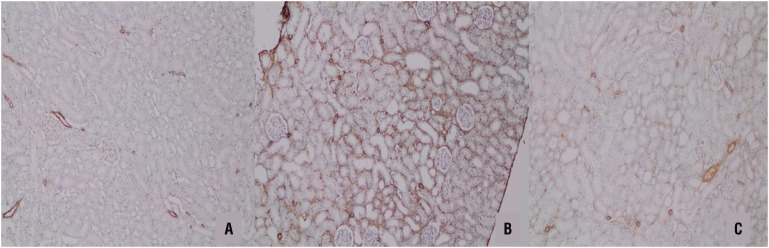
A) α-SMA staining was not observed in the tubules of the sham group. B) Moderate -SMA positivity was observed in more than 50% of the tubules in the UUO group. C) mild -SMA positivity was observed in 25-50% of the tubules in the OPZ-treated group (-SMAx100).

UUO caused significant interstitial fibrosis in rats that received no treatment (group 3). The percentage of the area of interstitial fibrosis in the rats with UUO that received no treatment was significantly greater than that of rats with UUO that received OPZ (group 4) (Figures 3A-C). These changes are summarized in Table-2. Expressions of α - SMA in immunohistochemistry showed that the staining positivity decreased in the tubules of the OPZ - treated group (Figures 4A-C).

**Table 2:** Scoring of tubular necrosis.

		Tubular necrosis				Interstitial fibrosis				Mononuclear cell infiltration			
	n	0	1	2	3	0	1	2	3	0	1	2	3
Sham	8	8	0	0	0	8	0	0	0	8	0	0	0
Sham+OP	8	8	0	0	0	8	0	0	0	8	0	0	0
UUO	8	0	1	3	4	0	1	4	3	0	1	2	5
UUO+OPZ*	8	0	6	2	0	2	5	1	0	1	5	0	2

**Score 0 =** no degeneration; **1 =** mild degeneration; **2 =** moderate degeneration; **3 =** severe degeneration

* Statistical significant difference from the UUO group and *P* < 0.05.

## DISCUSSION

In this study, we analyzed the protective effect of OPZ against renal fibrosis in a rat UUO model, a well - established in vivo model of renal fibrosis. Our study confirmed the protective role of OPZ through a quantitative examination of renal tissue damage after the induction of UUO in rats. To the best of our knowledge, this report is the first to show that OPZ has a preventive effect on functional, histological kidney injury caused by UUO.

The molecular mechanisms of tubulointerstitial fibrosis owing to UUO include several components such as inflammatory cell infiltration, fibroblasts and extracellular matrix production, and epithelial to mesenchymal transition. Among these, EMT has been of great interest to researchers during recent decades. EMT has been asserted as a functional and phenotypic change of epithelial cells that is reminiscent of mesenchymal cells reflecting a global process affecting adjacent cells (18). During the EMT process, expression of epithelial adhesion molecules such as E - cadherin is decreased whereas mesenchymal marker proteins such as α - smooth muscle actin (α - SMA) and N - cadherin are up - regulated. The EMT process has been viewed as a pathological predicator of renal fibrosis as myofibroblasts transformed from epithelial cells act as the main origin of ECM production (19). Moreover, the phenotype transformation of renal epithelial cells can cause dysfunction of the kidney, eventually resulting in glomerulosclerosis. Furthermore, accumulating lines of evidence support the concept that ROS affects EMT changes. Higher levels of ROS may aid the EMT process of epithelial cells and lead to fibrosis in the context of transcription factors and the multifunctional role of ROS in cellular signaling pathways (20, 21).

Nuclear factor - erythroid – 2 - related factor 2 (Nrf2) is a vital molecule of the endogenous antioxidant system that plays a central role in stimulating expression of various antioxidant - associated genes in cellular defense against oxidative stress. In the absence of oxidative stress, Nrf2 resides in the cytoplasm together with its repressor kelch - like ECH - associated protein 1 (KEAP1). Many agree that the upregulating in the production of antioxidant enzymes, which follows the detaching of Nrf2 from KEAP1 and subsequent movement into the nucleus, is a result of ROS overproduction or results from responses to electrophilic reagent treatment (22). Debates continue as to whether the EMT process is a real and direct contributor to renal fibrosis pathology in vivo (23, 24).

Based on the results showing the linkage between Nrf2 and TGFβ1 signaling, we hypothesized that Nrf2 could have a potential role in protecting the kidney in unilateral ureteral obstruction. It is believed that oltipraz, a prototype dithiolethione, might support the joining of the Nrf2 to the antioxidant response element (25).

We measured renal cortical E - cadherin, TGF - β1, hydroxyproline and NO levels biochemically as evidence of EMT. Except for the hydroxyproline level, all these markers were heightened in UUO rats and OPZ attenuated their levels. These results suggest that OPZ ameliorates the renal fibrosis by inhibiting these markers, which are the indicators of renal fibrosis. The possible explanation for the level of hydroxyproline not being significant among the groups might be the fact that the duration of UUO may be insufficient for observing the exact fibrotic changes in the kidney. It is probable that hydroxyproline, being a component of collagen, would be detected as increased in UUO kidney rats if they were examined one month or later after the obstruction date.

Additionally, pooled urine in UUO is markedly hypotonic (26). In theory, hypotonic cell growths could be caused by the hypotonic nature of pooled UUO urine. Increases in E - cadherin expressions and the induction of connective tissue growth factors (CTGF) were instigated through hypotonic media. It has been proposed that CTGF is an element of renal EMT via evidence of upregulation in UUO (27, 28).

We think that E - cadherin can be simultaneously upregulated in response to hypotonic stretch, suggesting that profibrotic activation of tubular cells might not require a ‘classical’ EMT process.

In this study, the histopathologic examination of kidneys showed severe and extensive damage in UUO rats with tubular necrosis and edema. This could be due to the formation of highly reactive radicals as a consequence of oxidative stress caused by UUO. The kidneys of the sham group showed normal histological features, but the UUO group revealed more extensive and marked tubular necrosis. On the other hand, the tubules from rats of the UUO + OPZ group were nearly normal in histological appearance except for slight desquamation and atrophy of the tubular epithelial cells. Also, expressions of α - SMA in immunohistochemistry showed that the staining positivity decreased in the tubules of the OPZ - treated group. Scientific evidence from the various field of diseases including pulmonary hypertension, liver ischemia / reperfusion injury yielded promising results on the potential effect of OPZ (29, 30). In a cell culture study, Atilano - Roque A et al. demonstrated that Nrf2 activating agent, OPZ had a beneficial effect on the viability of human kidney cells and expression of antioxidant and efflux transporter genes in proximal tubules which was exposed to cisplatin. Our results have similar findings compared to these studies. Given the similar pathophysiologic mechanisms, we may propose that OPZ is a promising agent for alleviating kidney injury in the presence of the ureteral obstruction.

We should address some potential limitations of this study. Firstly, we measured NO levels and reported that rats with UUO had increased levels of NO. However, our study lacks the measurement of NO isoforms including neuronal, endothelial, and inducible NOS. Secondly, although we exerted the conventional methods including the detection of the levels of molecules as mentioned earlier and histopathologic examination of renal tissue, we did not use quantitative RT - PCR, western blot, and immunofluorescence analysis to analyze further and demonstrate the expression of cell markers. Thirdly, although UUO is a well - accepted animal renal fibrosis model, it is criticized for the fact that the contralateral kidney may compensate for the deteriorated function of the obstructed kidney precluding meaningful renal function measurements (31). Lastly, whether the sensitivity and specificity of the markers used for EMT in our study are higher than some other EMT markers such as vimentin, cytokeratin, and β catenin is not known. Further studies on these markers will probably yield more insights to this debate.

## CONCLUSIONS

Taken all together, while the mechanism of actions remains unknown, our results show that OPZ plays a protective role in UUO - mediated renal fibrosis. However, further well - designed animal and clinical studies are needed to confirm our results.
